# Detecting health misinformation: A comparative analysis of machine learning and graph convolutional networks in classification tasks

**DOI:** 10.1016/j.mex.2024.102737

**Published:** 2024-04-28

**Authors:** Bharti Khemani, Shruti Patil, Ketan Kotecha, Deepali Vora

**Affiliations:** a Symbiosis Institute of Technology, Symbiosis International (Deemed University, Pune, India; b Symbiosis Centre for Applied Artificial Intelligence (SCAAI), Symbiosis Institute of Technology Pune Campus, Symbiosis International (Deemed University) (SIU), Lavale, Pune 412115, India

**Keywords:** Health, Misinformation detection, Covid-19, AI-based techniques, Natural language processing (NLP), Graph convolution networks (GCN), Graph Convolutional Network, Hybrid Graph Convolutional Network

## Abstract

In the digital age, the proliferation of health-related information online has heightened the risk of misinformation, posing substantial threats to public well-being. This research conducts a meticulous comparative analysis of classification models, focusing on detecting health misinformation. The study evaluates the performance of traditional machine learning models and advanced graph convolutional networks (GCN) across critical algorithmic metrics. The results comprehensively understand each algorithm's effectiveness in identifying health misinformation and provide valuable insights for combating the pervasive spread of false health information in the digital landscape. GCN with TF-IDF gives the best result, as shown in the result section.

• The research method involves a comparative analysis of classification algorithms to detect health misinformation, exploring traditional machine learning models and graph convolutional networks.
• This research used algorithms such as Passive Aggressive Classifier, Random Forest, Decision Tree, Logistic Regression, Light GBM, GCN, GCN with BERT, GCN with TF-IDF, and GCN with Word2Vec were employed. Performance Metrics: Accuracy: for Passive Aggressive Classifier: 85.75 %, Random Forest: 86 %, Decision Tree: 81.30 %, Light BGM: 83.29 %, normal GCN: 84.53 %, GCN with BERT: 85.00 %, GCN with TR-IDF: 93.86 % and GCN with word2Vec: 81.00 %
• Algorithmic performance metrics, including accuracy, precision, recall, and F1-score, were systematically evaluated to assess the efficacy of each model in detecting health misinformation, focusing on understanding the strengths and limitations of different approaches. The superior performance of Graph Convolutional Networks (GCNs) with TF-IDF embedding, achieving an accuracy of 93.86 %

## Graphical abstract

**Figure:**
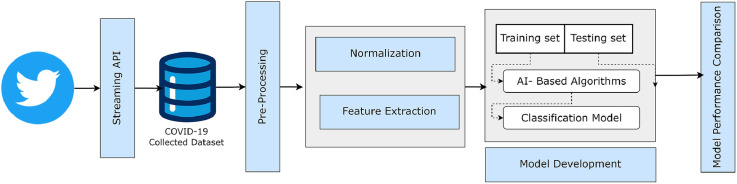

Specifications table

**Table:** 

Subject area:	Engineering
More specific subject area:	Health Misinformation, Graph Neural Network
Name of your method:	Graph Convolutional Network, Hybrid Graph Convolutional Network
Name and reference of the original method:	Khemani, B., Patil, S., Kotecha, K. A review of graph neural networks: concepts, architectures, techniques, challenges, datasets, applications, and future directions. J Big Data 11, 18 (2024). https://doi.org/10.1186/s40537-023-00876-4
Resource availability:	The code and data supporting the exemplary analysis are available in the Supplementary.

## Method details

This article aims to introduce a new deep learning technique based on graph neural networks. The method is a Graph convolutional network. Graph Convolutional Networks (GCNs) have emerged as a powerful tool for health misinformation detection, leveraging their ability to capture complex relationships within graph-structured data. Instead of using text data, we converted our text data into graphical form and applied a different model to calculate each model's performance metrics. By constructing a graph representation of the text corpus related to health information, GCNs capture structural dependencies between documents. This helps in understanding how different pieces of information are connected and how they collectively contribute to the context of health-related discussions. The effectiveness of GCNs in health misinformation detection can be further enhanced by integrating them with advanced embedding techniques. For example, combining GCNs with BERT (Bidirectional Encoder Representations from Transformers) or TF-IDF (Term Frequency-Inverse Document Frequency) embeddings allows the model to leverage graph-based relationships and rich contextual information within the text. Comparative studies often involve evaluating the performance of GCNs against traditional machine learning models, such as Passive Aggressive Classifiers, Random Forest, Logistic Regression, etc [1]. GCNs offer a promising approach to health misinformation detection by leveraging graph-based representations and integrating advanced embedding techniques. Their ability to capture structural dependencies and contextual understanding makes them valuable in the evolving landscape of health-related information dissemination, especially on platforms like social media.

In essence, social media has transformed public health outreach, enabling professionals to listen to the public, understand their needs, and effectively communicate critical information in the face of health emergencies [[2], [3]]. Since laypersons are typically called upon to play an active role in managing their health and that of others, as demonstrated most recently in the case of the COVID-19 pandemic, increasing the population's health literacy rates—including through the development of automated tools—becomes imperative in the current scenario of health misinformation dissemination [4]. The concepts of reliability, truthfulness, trustworthiness, credibility, integrity, etc., can be referenced in the state-of-the-art works described in the following section. These concepts can have different meanings depending on whether they refer to the information itself, the information source, the communication medium through which it is propagated, or other theoretical aspects [5]. The goal of the study is to close the knowledge gap that exists between misinformation judgment and health literacy. They use a survey methodology using tools and stimuli modified from earlier research. Through ordered probit regression, they discover that consumers' perceptions of the trustworthiness of health misinformation can be considerably reduced by raising health literacy. The empirical findings are used to discuss several implications [6]. Given the abundance of false material available online and the many parents who use the Internet to research vaccines, automated content classification can assist in directing users to trustworthy sources. We successfully trained and evaluated various classifiers on texts collected from pre-existing webpages using supervised machine learning. Moreover, phrases taken from websites pertaining to HPV vaccination were used to test the robustness of the best classifier. The classifier performed well in both scenarios, particularly when it came to identifying trustworthy information [7]. The study presents a Markov Random Field (MRF) model that takes into account the following factors: the statements' linguistic objectivity (identified by extracting linguistic-stylistic but also linguistic-emotional features), the user's reliability (based on engagement in the community and other information, such as socio-demographic factors), and the statements' reliability (about the medications to be taken and potential side effects, as reported in the Mayo Clinic dataset [8]). Even though the approach is highly disease-specific in this instance, [9] (one of the most significant online communities devoted to health) achieves a reasonable degree of accuracy. Another study on a particular disease examines people on social media endorsing proven-ineffective cancer treatments [10]. Integration of GCNs in Misinformation Detection: The integration of GCNs with machine learning and deep learning methods has shown promise in misinformation detection [11]. By constructing a graph representation of the text corpus, where documents are nodes and relationships between them are edges, GCNs can capture structural dependencies and enhance the model's understanding of document relationships. Several studies have successfully applied GCNs to detect misinformation, achieving improved performance compared to traditional approaches [12]. Shao proposes a graph convolutional network (GCN) approach by incorporating external knowledge graphs to see misinformation. They show that their method outperforms several other models on a benchmark dataset [13]. ``Fake News Detection: A Deep Learning Approach'' by Thorne et al. This paper proposed a deep learning approach for detecting fake news using text and image features. This paper proposed a method for detecting disinformation campaigns on social media using a combination of content-based and network-based features. They evaluated their approach on a dataset of news articles and social media posts related to the 2016 US presidential election [14]. PESTO involves a Posts/User Feature Encoder, which encodes the text and meta-features of a post/user into a dense vector, transformer model, Relational Graph Convolutional Network (RGCN) for user-follow network, and Fusion Network based on Self-Attention [15]. Microsoft Credibility Medical Web Corpus CLEF eHealth 2020 dataset and worked on Web Pages using the Vec4Cred model [16]. On the Covid-19 dataset, they used Text-Based tweets by applying the CNN model and got an accuracy of 85.98 % (ResNet+Linear classifier) [17].

**Fig. 1:**
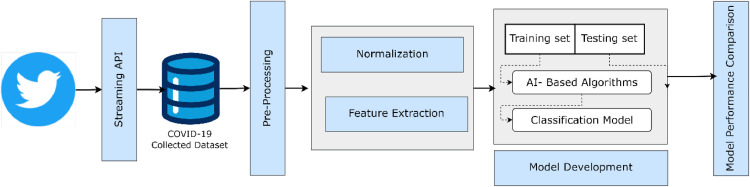
Steps for proposed architecture.

The steps of the proposed architecture of the suggested framework are shown in Fig. 1, which begins with collecting raw data via Twitter's streaming API and moves on to performance comparison and evaluation. We used a binary classification challenge to represent the COVID-19 misinformation issue. Our approach is predicated on a sizable dataset that human specialists gathered and annotated. The techniques employed in this work are predicated on the idea that every tweet has unique characteristics. We review how we collected and categorized the COVID-19-related Twitter data in the following subsections. Next, we review several feature categories employed to evaluate the integrity of the content in each tweet.

**Fig. 2:**
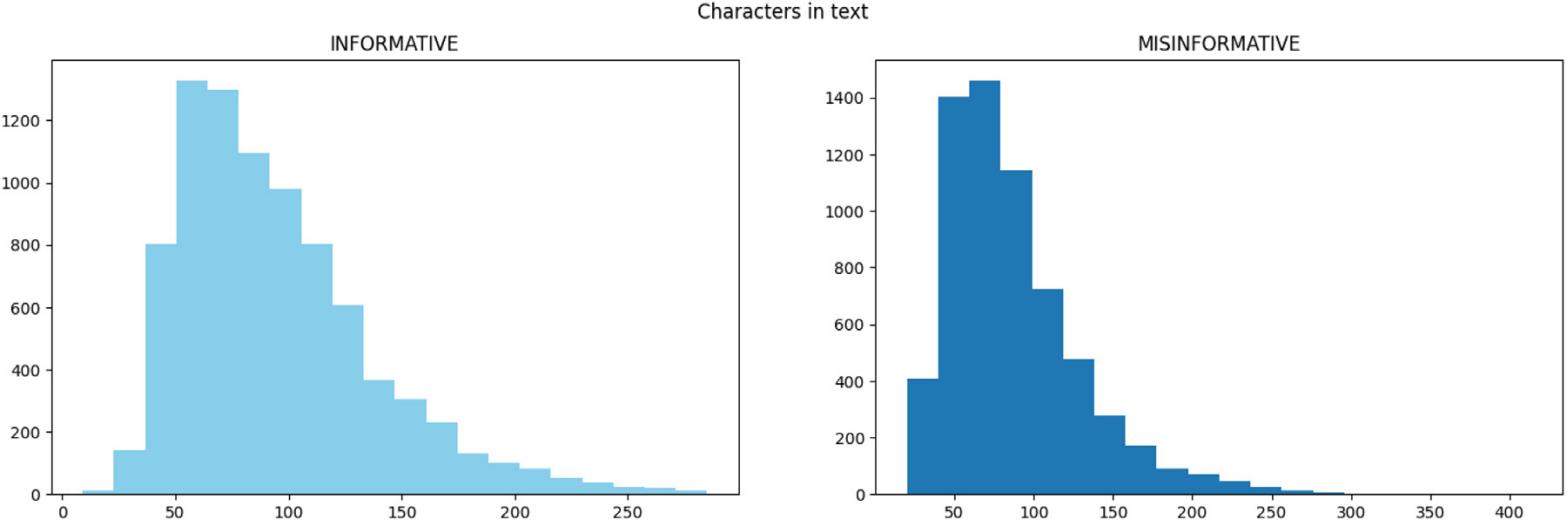
Reliable and unreliable tweets with the number of characters in tweets.

Dataset Description and Streaming API: The two columns in the dataset, which includes 15,635 tweets, are tweets and a combination of reliable and unreliable labels. The dataset is labeled using the official Google web pages. Here in the dataset are 8958 reliable Labels and 6677 unreliable labels. We generated a dataset of tweets using Twitter's streaming API to carry out our experiment. Our search used COVID-19, health, healthcare, cancer, Ayurveda, and misinformation-related hashtags and keywords. The tweets were identified, annotated, and classified based on the type of tweet. Fig. 2 shows the Reliable and unreliable tweets with the number of characters in tweets.

Twitter's streaming API allows real-time access to tweets based on specific keywords, hashtags, or user criteria. For gathering COVID-19-related data, relevant keywords such as ``COVID-19,'' ``coronavirus,'' ``pandemic,'' etc., were used to filter tweets. The streaming API continuously retrieves tweets that match the specified criteria, providing a constant flow of real-time data related to COVID-19 discussions on Twitter. Data collection parameters such as language preferences, geographic locations, and tweet types (e.g., original tweets and retweets) were configured to refine the dataset based on research requirements.

The dataset was gathered from multiple real-time Twitter data sources. The dataset undergoes data pre-processing to eliminate empty columns, URLs, and punctuation. Tokenization breaks the longer text into words or short lines after text preparation. The alternatives are extracted from the text in a series of steps. The progressive metric capacity unit learning algorithms get the removed options, after which they coach the model. Analytical criteria are commonly employed to evaluate the efficacy of metric capacity unit algorithms. Table 1 describes some rows of our dataset. Data collection, applying pre-processing steps for data cleaning, annotating tweets for classification purposes, and creating a validated dataset for further analysis and model training in the context of COVID-19-related misinformation detection.

**Table 1:** Some samples of dataset.

	Tweet	Label
0	Bill Gates profits from vaccination and wants…	Unreliable
1	Politically Correct Woman Almost Uses Pandemic…	Unreliable
2	Coronavirus Response A Chaotic Disaster	Unreliable
3	Clearly the Obama administration did not leave…	Unreliable
4	Retraction Hydroxychloroquine or chloroquine w…	Unreliable

**Fig. 3:**
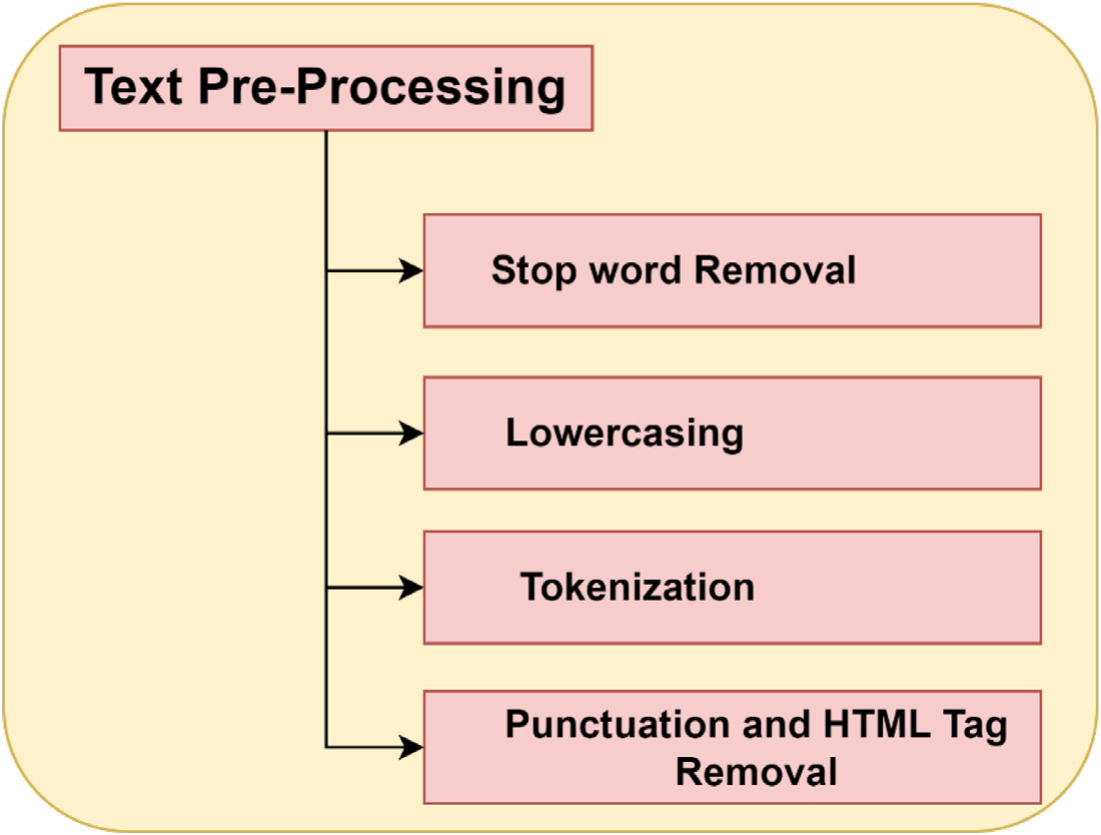
Pre-Processing Steps.

**Pre-Processing:** The study utilized an uncontaminated, raw dataset. It will be necessary to clean up the dataset by eliminating some unnecessary symbols that impact the final classification of the article. As part of the pre-processing step, URLs are eliminated from the dataset to eliminate unnecessary characters such as punctuation marks. Text is divided into a set of tokens through a process called tokenization. The fake news dataset's lengthy sentences have been tokenized into smaller words or tokens. The data preprocessing step are shown in Fig. 3.

1. Lowercasing: Convert all text to lowercase or uppercase to ensure consistency in text processing. This prevents the model from treating ``word'' and ``Word'' as different tokens.
2. Stop Word Removal: Remove common words (stop words) like ``the,'' ``is,'' ``in,'' etc., that often carry little meaning and can be safely discarded for many NLP tasks.
3. Noise Removal: Remove any irrelevant or noisy elements from the text, such as HTML tags, special characters, or punctuation, depending on the specific task and dataset.
4. Tokenization: Divide the text into individual words or tokens. Tokenization breaks a text into its basic units, usually words or sub words (sub word tokenization is common for languages like Chinese or Japanese).

Table 2 shows the Output after applying Pre-proessing step.

**Table 2:** Output of Pre-proessing step.

Tweet	Cleaned_tweet	Tweet length	Word Count	Label Encoded
The CDC currently reports 99,031 deaths. In general discrepancies death counts different sources small explicable. The death toll stands roughly 100,000 people today.	Cdc currently report death general discrepancy death count different source small explicable death toll stand roughly people today	167	26	1
States reported 1121 deaths small rise last Tuesday. Southern states reported 640 deaths. https://t.co/YASGRTT4ux	State reported death small rise last tuesday southern state reported death tcoyasgrttux	117	18	1
Politically Correct Woman (Almost) Uses Pandemic Excuse Not Reuse Plastic Bag https://t.co/thF8GuNFPe # coronavirus # nashville	politically correct woman almost us pandemic excuse reuse plastic bag tcothfgunfpe coronavirus nashville	131	20	0
# IndiaFightsCorona : We 1524 # COVID testing laboratories India 25th August 2020 36,827,520 tests done : @ ProfBhargava DG @ ICMRDELHI # StaySafe # IndiaWillWin https://t.co/Yh3ZxknnhZ	Indiafightscorona covid testing laboratory india th august test done profbhargava dg icmrdelhi staysafe indiawillwin tcoyhzxknnhz	185	29	1

**Normalization and Feature Extraction:** We used NLTK library of NLP, which used Stemming and lemmatization. They are text normalization techniques. Stemming reduces words to their root or stem form, while lemmatization maps words to their base or dictionary form. Both methods help reduce variations in word forms and simplify text analysis.

**Fig. 4:**
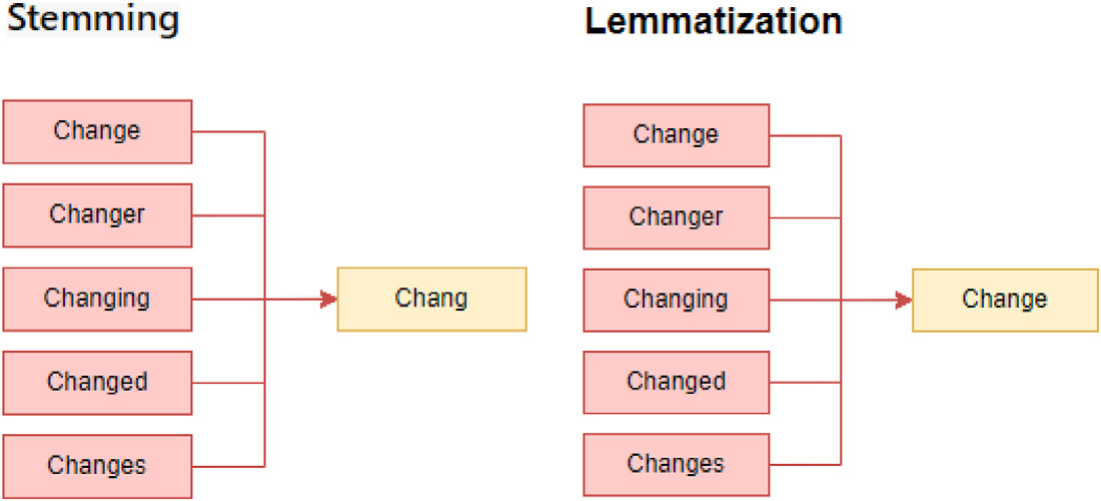
Example of stemming and lemmatization.
(2) Text Vectorization: Represent entire documents or sentences as numerical vectors. Standard methods include TF-IDF (Term Frequency-Inverse Document Frequency) and Bag of Words (BoW). We use TF-IDF vectorization to transform the text data into numerical features. Fig. 5 gives the output after applying feature extraction techniqus.

(1) Stemming and Lemmatization: Reduce words to their base or root form to capture their essential meaning. They are stemming and lemmatization, which help reduce dimensionality and improve text analysis. Stemming is more aggressive and may result in non-words, while lemmatization produces valid words. Example is shown in Fig. 4.

**Fig. 5:**
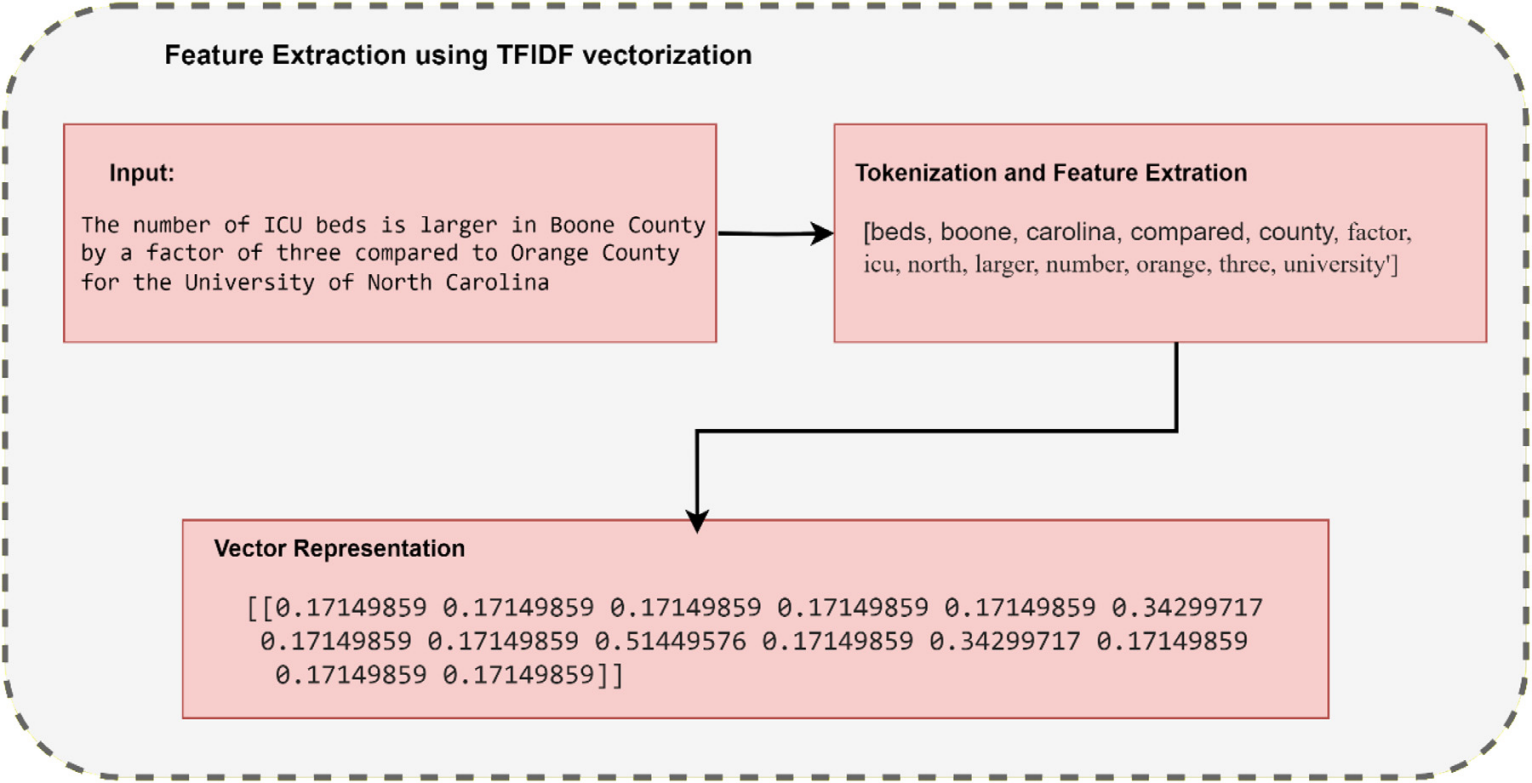
Output after applying Feature Extraction using TF-IDF technique.

These normalization and feature extraction procedures were crucial in converting raw text data into structured numerical representations suitable for machine learning analyses. They captured semantic meaning, context, and relationships within the text, enabling our algorithms to accurately perform classification and sentiment analysis tasks.

Feature extraction involves transforming the text into numerical features that GNNs can work with. Methods like Term Frequency-Inverse Document Frequency (TF-IDF)) and word embedding (Word2Vec) can be used to represent words as vectors. Mathematical explanations of TF-IDF (Term Frequency-Inverse Document Frequency) and Word2Vec are implemented to represent words as vectors. TF-IDF combines TF and IDF to evaluate the importance of a term in a specific document relative to the entire corpus.

## Mathematical representation

(1) Term Frequency (TF) for a term t in a document d is calculated as:
  (I) $$TF\left(t,d\right)=\frac{Number\;of\;times\;term\;t\;appears\;in\;document\;d}{Total\;number\;of\;terms\;in\;document\;d\;}$$
(2) Inverse Document Frequency (IDF) for a term t in a corpus D is calculated as:
  (II) $$IDF\left(t,D\right)=\log\frac{Total\;number\;of\;documents\;in\;corpus\;D}{number\;of\;documents\;containing\;term\;t\;in\;corpus\;D}$$
(3) **TF-IDF (Term Frequency-Inverse Document Frequency)**:
  (III) TF−IDF(t,d,D)=TF(t,D)*IDF(t,D)

d represents a specific document within a collection of documents. And D represents the total number of documents in the corpus.

Implementation:

Each term in a document is represented by its TF-IDF score in a vector space. The vector representing a document contains TF-IDF scores for all terms in the vocabulary. The TF-IDF vectors are typically normalized to unit length for better comparison and computation.

Word2Vec: Word2Vec is a popular word embedding technique representing words as dense vectors in a continuous vector space. It uses neural networks to learn word embeddings based on contextual information from large text corpora. Word2Vec vectors represent words in a constant vector space where similar words are closer in the vector space, capturing semantic similarities. TF-IDF and Word2Vec are mathematically implemented to represent words as vectors by leveraging statistical measures (TF-IDF) and neural network architectures (Word2Vec) to capture word importance and semantic relationships, respectively.

In TF-IDF (Term Frequency-Inverse Document Frequency), punctuation marks are typically treated as separate "words" during the text processing stage. This is because punctuation can carry important semantic and syntactic information in natural language text, especially when detecting misinformation where subtle cues like exaggerated claims or misleading statements may be conveyed through punctuation. Similarity measures between word vectors can be employed to identify deceptive language patterns or abnormal usage of words in misinformation contexts.

**Model Development:** After pre-processing the combined health-related dataset, the data was split into two distinct sets: the training set and the testing set. In the training set, which constitutes a significant portion of the data, the ratio of splitting data is 20 % for testing and 80 % for testing utilized for training the machine learning model. It consists of labeled examples of pre-processed text data and is the foundation for the model to learn sentiment patterns and relationships. The testing set, on the other hand, remains unseen during the training process and is reserved for evaluating the model's performance. This separation allows for assessing the model's ability to generalize and make predictions on new, unseen data. It is crucial in estimating the model's accuracy and other performance metrics, ensuring its reliability in real-world applications.

Mathematically, GNN operations involve iterative updates of node embeddings using message-passing algorithms, where each node aggregates information from its neighbors based on learned edge weights and node features. The specific equations and algorithms depend on the GNN architecture and message-passing scheme (e.g., sum, mean, attention). The mathematical support of the relation between text, vectors, and graphs in the context of GNNs for text analysis and misinformation detection lies in integrating normalized numerical features into a structured graph representation, followed by applying GNN operations for learning and inference tasks.

## Performance metrics

We computed several measures, including as accuracy, precision, recall, F1 score, and ROC-1) AUC score, to assess the effectiveness of our model. These indicators offer several viewpoints on the performance of your model. The following describes each metric's normal calculation and meaning:

(1) Accuracy: The percentage of correctly identified samples relative to the total number of samples is known as accuracy. It's a typical measure of the overall performance of the model.
  (IV) $$Accuracy=\frac{Number\;of\;correct\;predictions}{Total\;number\;of\;predictions}$$
(2) Precision: The percentage of true positive predictions—that is, accurately predicted positive samples—among all positive predictions is known as precision. It evaluates how accurate positive forecasts are.
  (V) $$Precision=\frac{True\;Positives}{True\;Positives+False\;positives}$$
(3) Recall (Sensitivity): Recall measures the proportion of true positive predictions from all actual positive samples. It assesses the ability of the model to capture all positive samples.
  (VI) $$Recall=\frac{True\;Positives}{True\;Positives+False\;Negatives}$$
(4) F1 Score: The F1 score is the harmonic mean of precision and recall. It balances precision and recall, making it useful when the class distribution is imbalanced.
  (VII) $$F1\;Score=\frac{2*\left(Precision*Recall\right)}{\left(Precision+Recall\right)}$$

## AI-Based algorithms: deep learning algorithms

**Fig. 6:**
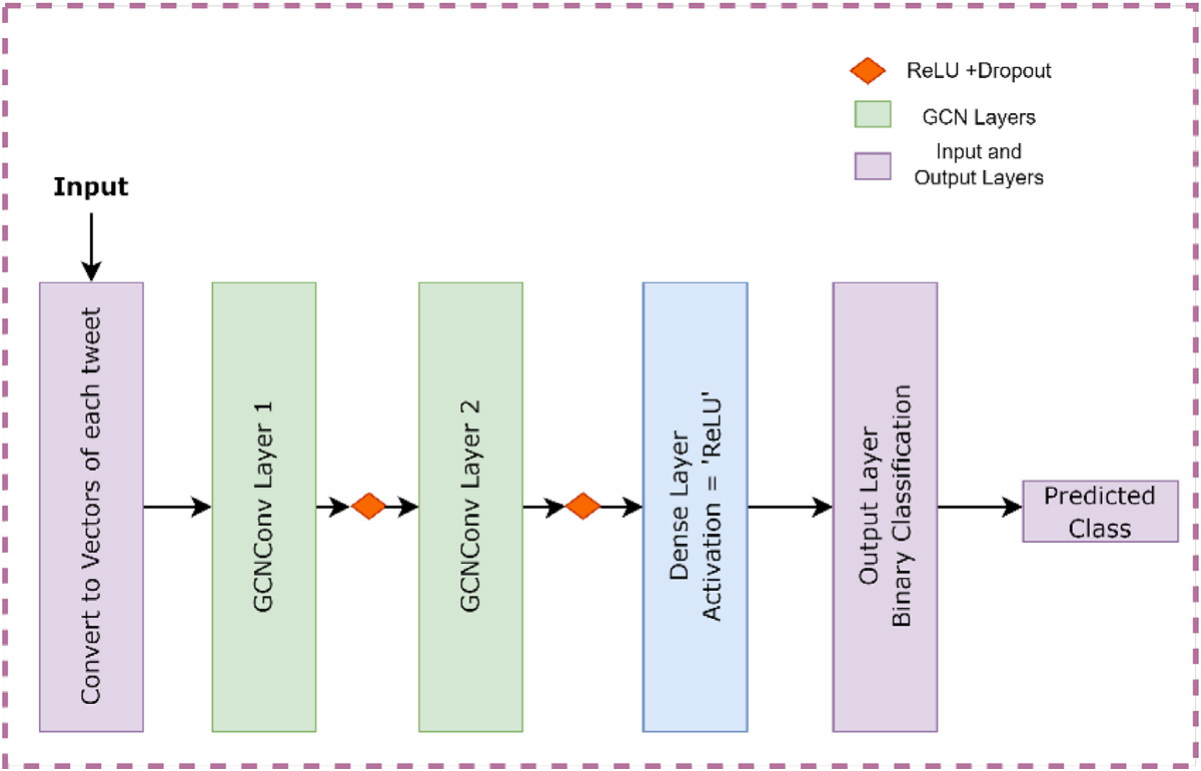
Graph convolution model.

Graph Convolutional Network (GCN): A graph is a type of data comprised of nodes connected by edges that could be directed. Every node has a set of features, and the edges between them represent the interactions between them. In a traditional GNN, messages are passed between nearby nodes via the edges. It makes it logical that the neural network encodes the data that is transferred from one node to its connected neighbors. A node's representation in a GCN is created by aggregating messages from all its neighbors to the current node, regardless of layer. A vector representation, or embedding representation, that describes the neighborhood graph structure and the node's feature information can be obtained for each node after several message transmission iterations. The architecture of GCN model is shown in Fig. 6.

Graph Convolutional Networks (GCN) with BERT: Graph Convolutional Networks and BERT (Bidirectional Encoder Representations from Transformers) are two distinct types of neural network architectures designed for different tasks. Here, we combine these two architectures in specific scenarios where your data exhibits graph-like structures and textual information. This integration can be beneficial in applications where there's a need to capture both the relational information in a graph and the contextual information embedded in text.

Graph Convolutional Networks (GCN) with TF-IDF: In this, we first construct a graph by representing our as a graph, where nodes represent entities, and edges denote relationships between entities. After that, features for each node in the graph are extracted. These features might include information about the node, neighbors, and other relevant attributes. For associated text data nodes, apply TF-IDF to convert the textual information into numerical vectors. Each node with text would have a TF-IDF vector representing its content. Combine the learned node embeddings from the GCN with the TF-IDF vectors through concatenation and averaging techniques.

## Method validation

**Fig. 7:**
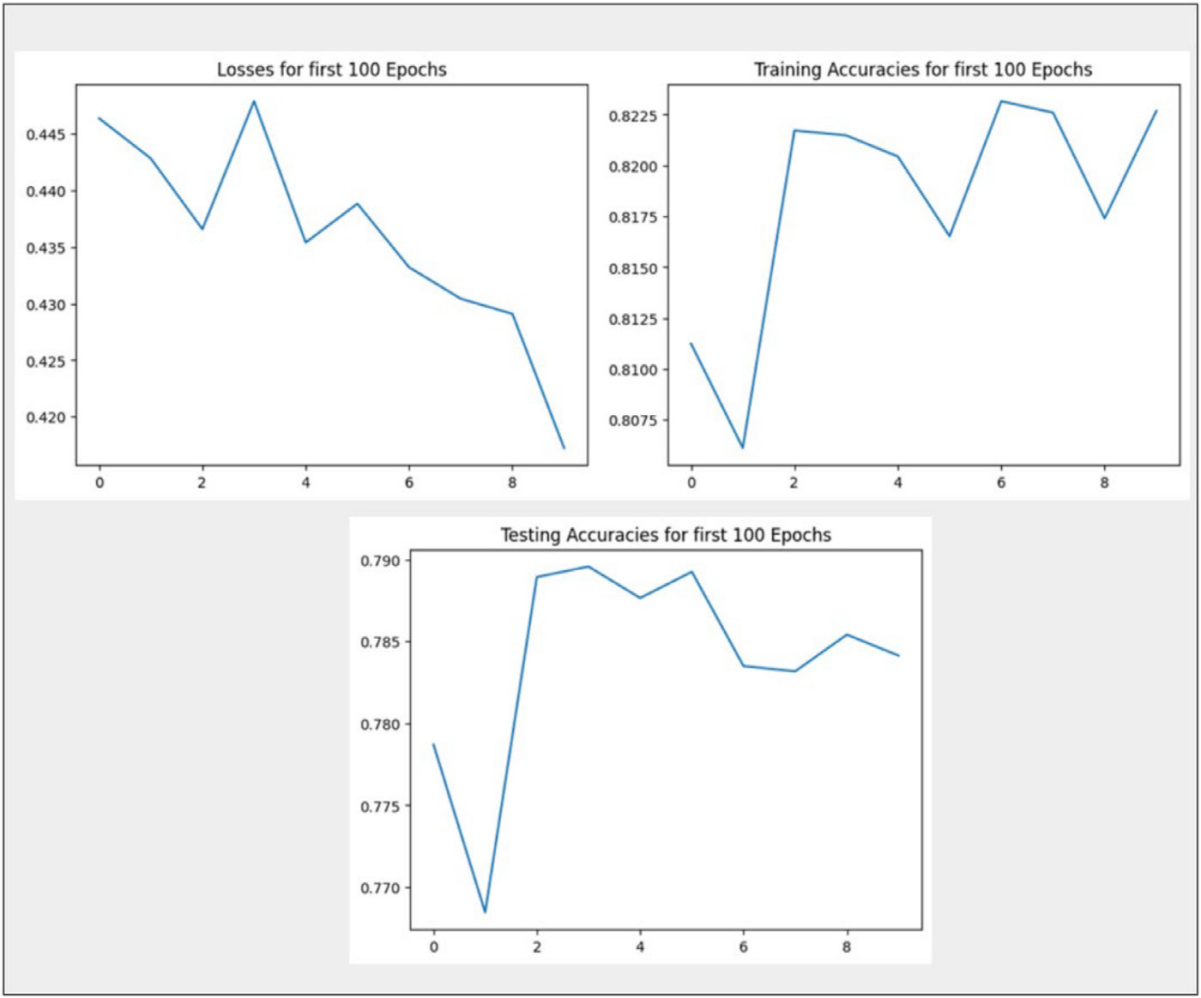
Output of thevGCN technique (Top left: loss function for 100 epochs, top right: training accuracies for first 100 epochs, and bottom: testing accuracies for first 100 epochs.).

Table 3 describes the performance metrics for different machine learning classifiers, including Graph Convolutional Networks (GCN), across various algorithms and embedding techniques. The performance of the models varies across different classifiers and embedding methods. GCN combined with BERT and TF-IDF outperforms other models, achieving higher accuracy and precision. The choice of the embedding technique seems to have a substantial impact on the model's performance. Support values indicate the number of instances considered for evaluation. Table 3 suggests that the combination of GCN with BERT and TF-IDF performs well on the given task, but the best model choice depends on specific requirements and trade-offs between precision, recall, and accuracy. Fig. 7 shows the output of the GCN technique. Top left: Loss Function for 100 Epochs, Top Right: Training Accuracies for First 100 Epochs, and Bottom: Testing Accuracies for First 100 Epochs.)

**Table 3:** Comparative analysis of ML and GCN techniques with different performance metrics.

	Algorithm								
Performance Metric	ML Classifiers					GCN			
	Passive Aggressive Classifier	Random Forest Class	Decision Tree Classifier	Logistic Regression	Light GBM Classifier	GCN	With BERT	With TF-IDF	With Word2Vec
Accuracy	85.75	86.00	81.30	83.29	84.53	85.00	88.86	93.86	81.00
Precision	86.00	86.00	81.00	83.00	85.00	83.00	88.00	94.00	82.00
Recall	85.00	86.00	81.00	83.00	85.00	82.00	88.00	94.00	82.00
F1-score	85.00	86.00	81.00	83.00	85.00	83.00	89.00	94.00	81.00

Advantages of Graph Neural Networks (GNNs) over Traditional Neural Networks:

(1) Handling Graph-structured Data: GNNs are specifically designed to work with data represented in graph structures, where entities (nodes) are interconnected through relationships (edges) [18].
(2) Capturing Structural Dependencies: Unlike CNNs that operate on regular grid-like structures (e.g., images), GNNs can capture complex dependencies and relationships between entities in a graph. This is crucial for tasks where understanding the context and connections between elements is paramount, as is often the case in detecting health misinformation where textual content may exhibit nuanced relationships.
(3) Adaptive Learning: GNNs excel at adaptive learning by aggregating information from neighboring nodes in a graph. This adaptability allows GNNs to learn and update node representations based on local and global graph structures, enabling them to capture context and semantics effectively.

**Fig. 8:**
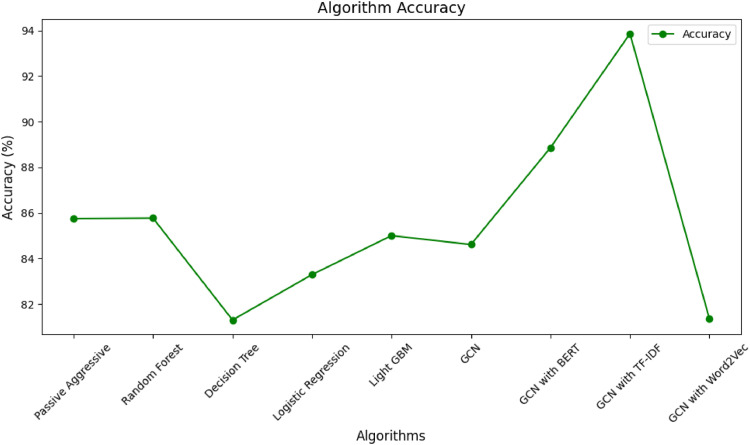
Line chart for all algorithms with accuracy metric.

Fig. 8 gives the output of all algorithms with accuracy metrics. The choice of classifier and feature representation significantly impacts the model's performance. The GCN, especially when combined with TF-IDF embeddings, appears to be the most effective task. The results highlight the importance of selecting appropriate models and embeddings based on the nature of the data and the task at hand. GCN with TF-IDF embeddings achieves even higher accuracy than GCN with BERT. This could be because TF-IDF captures document-level information effectively, and the graph structure enhances the model's ability to understand relationships between documents. GCN with Word2Vec embeddings achieves the lowest accuracy among the GCN variants. Word2Vec provides word-level embeddings, and in this case, it might not capture the contextual information as effectively as BERT or the document-level information as effectively as TF-IDF for the given task.

**Fig. 9:**
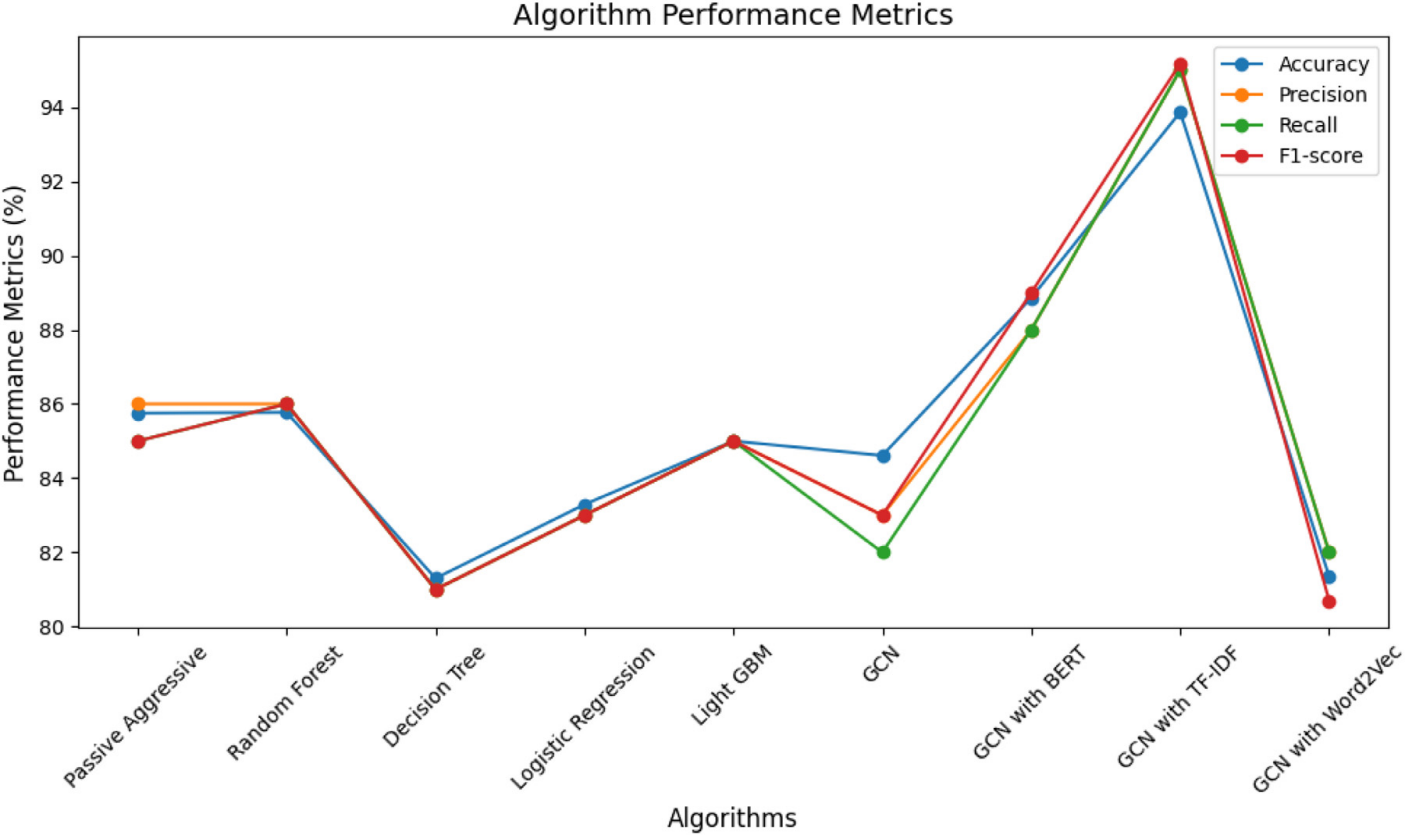
Comparative analysis of all algorithms with all performance metrics.

Fig. 9 provides the details of all algorithms with all performance metrics. The additional metrics (Precision, Recall, F1-score, and Support) offer a more comprehensive view of the classifiers' performance. GCN with TF-IDF embeddings demonstrates the highest precision at 94.00 %, showcasing its effectiveness in correctly identifying positive instances. GCN with TF-IDF embeddings shows the highest recall at 94.00 %, indicating its point in capturing almost all positive models. GCN with TF-IDF embeddings demonstrates the highest F1-score at 89.00 %, showcasing its effectiveness in achieving a balanced performance. GCN with TF-IDF embeddings consistently outperforms other models across all metrics, indicating its suitability for the given task. Traditional machine learning classifiers, such as Random Forest and Logistic Regression, perform well but with slightly lower accuracy than the GCN with TF-IDF. The choice of embeddings (BERT, TF-IDF, Word2Vec) significantly impacts the performance of the GCN.

Summary: Rationale for Choosing GNN as the Deep Learning Technique:

(1) Graph-based Representation of Textual Data: Our study involves converting textual data related to health information into a graph-based representation. This decision was motivated by the inherent relational nature of health-related content, where understanding the connections between medical terms, concepts, and contextual information is crucial for detecting misinformation. [19]
(2) Utilizing Structural Dependencies: By leveraging GNNs, we aimed to exploit the structural dependencies in the graph representation of health-related textual data. GNNs are well-suited for capturing these dependencies and extracting meaningful patterns that contribute to the detection of misinformation.
(3) Integration with Embedding Techniques: GNNs can be integrated with advanced embedding techniques such as TF-IDF and Word2Vec, enhancing their ability to capture semantic relationships and contextual information within the graph. This integration further boosts the performance of GNNs in tasks like health misinformation detection.
(4) Comparative Performance Evaluation: Our decision to choose GNNs was also informed by a comparative analysis of different deep learning techniques, where GNNs demonstrated superior performance in terms of accuracy, precision, recall, and F1-score compared to traditional neural networks like CNNs or fully connected networks.

Future scope: Our feature importance analysis section addresses your concern by offering a detailed examination of important features, assessing consistency across different techniques, providing interpretability and model insights, conducting comparative analyses, and discussing implications for future research. Also, we will work on summarizing the computational costs of various ML and GCN techniques.

## Ethics statements

Not applicable.

## CRediT authorship contribution statement

**Bharti Khemani:** Conceptualization, Methodology, Validation, Data curation, Writing – original draft, Visualization, Investigation, Software, Writing – review & editing. **Shruti Patil:** Conceptualization, Methodology, Validation, Data curation, Writing – original draft, Visualization, Investigation, Supervision, Funding acquisition. **Ketan Kotecha:** Visualization, Investigation, Supervision, Funding acquisition. **Deepali Vora:** .

## Declaration of competing interest

The authors declare that they have no known competing financial interests or personal relationships that could have appeared to influence the work reported in this paper.

## Data Availability

Data will be made available on request.
